# Localization accuracy of 6‐second CBCT for lung IGRT with various breathing patterns

**DOI:** 10.1002/acm2.70130

**Published:** 2025-05-29

**Authors:** Jihye Koo, Gage Redler, Vladimir Semenenko, Stephen A. Rosenberg, Emily Keit, Jacqueline M. Andreozzi

**Affiliations:** ^1^ Department of Radiation Oncology H. Lee Moffitt Cancer Center and Research Institute Tampa Florida USA

**Keywords:** 4DCT, CBCT, IGRT, localization, respiratory motion

## Abstract

**Purpose:**

The 6‐second cone beam computed tomography (CBCT) acquisition of the Ethos HyperSight (Varian Medical Systems, Inc. Palo Alto, CA, USA) on‐board imaging system offers benefits, but could be too fast to accurately capture an average target position in a free‐breathing lung cancer patient. This study aimed to ascertain whether a 6‐second acquisition is appropriate for regularly breathing patients with varying respiration periods. Additionally, breathing patterns that could lead to clinically impactful inaccuracies for image‐guided radiation therapy (IGRT) shifts were investigated.

**Methods:**

Nine regular (sinusoidal) breathing patterns with different respiration rates (8–20 breaths‐per‐minute) and amplitudes, along with five irregular breathing patterns including a gasp/cough scenario, were tested using a respiratory motion phantom with a 3 cm diameter spherical target. Once the phantom was aligned using the external chassis to remove any default shifts, the 6‐second CBCTs with Acuros reconstruction were acquired. Rigid registrations were performed using the 4DCT simulation average/untagged reconstructed image as reference to align to target (simulating clinical lung IGRT setup). Sixty‐second CBCTs were also tested to verify the average offsets with conventional practice. The IGRT shifts were compared to 5 mm, a PTV margin typically used for lung tumors.

**Results:**

Minimal (<1.0 mm) shifts were observed for all regular breathing patterns with both 6‐second and 60‐second CBCT acquisitions. For moderately irregular breathing patterns and the gasp/cough scenario, all shifts were less than 5 mm. Shifts larger than 5 mm were observed in highly irregular breathing patterns, with both 6‐second (14%) and 60‐second (24%) CBCT acquisitions. Statistical tests showed no significant differences (*p *> 0.05) between the sizes of shifts made with the two CBCT durations.

**Conclusion:**

The 6‐second CBCT can effectively and reliably localize a mobile target for regular and moderately‐irregular breathers. Cautions should be used for highly irregular breathers, regardless of the duration of CBCT acquisition.

## 1 INTRODUCTION

Cone‐beam computed tomography (CBCT) is essential in image‐guided radiation therapy (IGRT) for verifying patient positioning and ensuring accurate tumor localization before treatment.^[^ ^1^, ^2^, ^3^, ^4^ ^]^ However, conventional CBCT systems on‐board clinical linear accelerators, which typically require about one minute to acquire images, present challenges ^[5^ ^]^ such as motion artifacts and increased radiation exposure due to the prolonged scan times. While increased exposure is not a significant clinical concern due to its small proportion relative to the treatment dose, motion artifacts not only compromise image quality but also hinder the accuracy of patient setup by potentially providing misleading reassurance, thereby affecting the precision of radiation delivery ^[6^ ^]^. This is particularly concerning in lung cancer, where the tumor is subject to motion due to respiration ^[^ ^7^, ^8^ ^]^.

In an effort to overcome the limitations of conventional CBCT, a novel kV imaging system with a fast CBCT has been recently introduced. The Ethos HyperSight imaging solution (Varian Medical Systems, Inc., Palo Alto, CA, USA) offers discrete 6‐second and 60‐second CBCT acquisition modes. These modes, combined with advanced features such as an enhanced detector panel and a sophisticated reconstruction algorithm ^[^ ^9^, ^10^ ^]^, achieve diagnostic‐level image quality that supports a variety of clinical applications, including enabling direct dose calculation on CBCT and online adaptive radiotherapy. By reducing the imaging time and leveraging the enhanced reconstruction algorithm, the system aims to minimize the risk of motion artifacts, thereby improving the clarity and accuracy of the images acquired. Recent studies have explored various aspects of the HyperSight system, including improvements in image quality and geometric accuracy ^[^ ^11^, ^12^, ^13^, ^14^ ^]^, as well as its feasibility for adaptive radiotherapy workflows, including dose calculation accuracy ^[^ ^15^, ^16^, ^17^, ^18^, ^19^, ^20^, ^21^ ^]^. While these studies provide valuable insights into the broader applications of the HyperSight system, the accuracy of the 6‐second CBCT in tumor localization under different respiratory conditions remains an area requiring further investigation. Zhao et al. ^[14^ ^]^. investigated various aspects of the HyperSight system, including tumor localization accuracy, across a range of respiratory patterns. While this study provides a broad assessment of the system, further research is needed to assess the clinical feasibility of 6‐second CBCT for lung IGRT patient setup, particularly in cases of irregular breathing patterns.

While the fast scan mode has the potential to enhance the accuracy of tumor localization, the narrow acquisition window raises concerns about the system's ability to fully capture the complete range of target motion in lung cancer patients. The 60‐second CBCT, with its longer acquisition time, benefits from averaging out target motion, resulting in a more consistent image that captures the entire movement cycle of the tumor and will match a reference slow‐pitch 4DCT average/untagged reconstructed image. In contrast, for an average breathing cycle period of around 4‐s (corresponding to a respiratory rate of 15 bpm) the 6‐second scan will capture only 1.5 breathing cycles, which may provide a limited snapshot, potentially missing portions of the tumor's range of motion. This limitation could lead to daily geometric misalignment, thus underdosing the target volume and/or overdosing normal tissue, and potentially compromising treatment outcomes.

Given these considerations, this study aimed to evaluate whether the HyperSight system's 6‐second CBCT could reliably capture the full range of tumor motion and maintain the precision necessary for effective lung IGRT setup under various respiratory conditions. Both ideal (i.e., regular) and non‐ideal (i.e., irregular) respiratory patterns were investigated.

## 2 MATERIALS AND METHODS

### 2.1 Respiratory motion phantom setup

**FIGURE 1:**
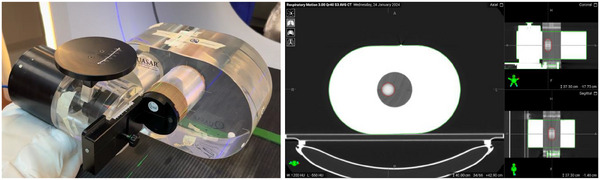
QUASAR respiratory motion phantom with lung insert containing a 3 cm spherical target (left) and a 4DCT average reconstruction of the phantom (right). The spherical target is contoured in red on the CT.

A QUASAR Respiratory Motion Phantom (Modus Medical Devices, London, Ontario, CA) was used to simulate lung tumor motion. The phantom has a cylindrical insert with density similar to lung tissue, with an off‐centered 3 cm diameter spherical target of higher density mimicking a tumor. The insert moves along the longitudinal axis, following programmed breathing patterns (Figure 1).

A series of respiratory patterns were tested, including nine regular sinusoidal breathing cycles with varying respiration rates (8–20 breaths per minute) and amplitudes (2.5–20.0 mm) (Table 1). For all breathing patterns, the motion amplitude is defined as peak‐to‐center displacement. These parameters were selected to represent the clinically relevant range of breathing patterns observed in patients undergoing lung IGRT. Notably, very long respiratory cycles beyond 8‐s per breath were excluded as they are rarely encountered in clinical practice since patients with diseases tend to breathe at a higher rate than those without ^[^ ^22^, ^23^, ^24^ ^]^. In addition, five irregular breathing patterns, which include variations such as asymmetry in inhalation/exhalation duration, amplitude fluctuations, or sudden changes in motion, were tested, including a scenario simulating a gasp or cough (Table 2). Gasping and coughing produce nearly identical sharp peaks in breathing cycles, so a single pattern with a sharp peak was used to represent both scenarios. The irregular breathing patterns were chosen to reflect clinically relevant challenges seen in daily patient setup.

**TABLE 1:** Regular breathing patterns with variable breaths‐per‐minute (bpm) and amplitudes (mm).

	Respiration rate (bpm)	Period (s)	Amplitude (mm)	CBCT acquisition time (s)	Shifts (mm)	RMSE (mm)
Respiration rate dependency	20	3.0	13.0	60	−0.2	
				6	−0.5 ± 0.0	0.5
	15	4.0	13.0	60	−0.2	
				6	−0.1 ± 0.1	0.1
	10	6.0	13.0	60	0.1	
				6	0.0 ± 0.1	0.1
	8	7.5	13.0	60	0.2	
				6	0.2 ± 0.0	0.2
Amplitude dependency	15	4.0	15.0	60	0.5	
				6	0.4 ± 0.0	0.4
	15	4.0	10.0	60	−0.3	
				6	−0.2 ± 0.1	0.2
	15	4.0	7.5	60	−0.5	
				6	−0.5 ± 0.0	0.5
	15	4.0	5.0	60	−0.4	
				6	−0.5 ± 0.1	0.5
	15	4.0	0.25	60	−0.1	
				6	−0.1 ± 0.0	0.1

*Note*: Shift values are mean ± one standard deviation. *N* = 1 and 3 for 60‐ and 6‐second CBCTs, respectively.

**TABLE 2:** Irregular breathing patterns.

		Reference 4DCT breathing pattern for rigid registration	6s CBCT		60s CBCT		p‐value
			Shifts (mm)	RMSE (mm)	Shifts (mm)	RMSE (mm)	
Moderately irregular patterns	(1)	Regular	0.4 ± 1.2	1.2	1.6 ± 1.2	2.0	0.055
	(2)	Regular	−3.0 ± 1.5	3.3	−1.7 ± 1.3	2.1	0.055
Highly irregular patterns	(1)	Regular	3.2 ± 2.0	3.7	4.2 ± 2.1	4.7	0.759
		Irregular	1.1 ± 2.8	3.0			
	(2)	Regular	4.7 ± 2.2	5.2	6.1 ± 0.9	6.2	0.164
		Irregular	2.3 ± 1.1	2.5			
Gasp/Cough		Regular	−0.3 ± 1.1	1.2	−0.1 ± 0.7	0.7	0.988

*Note*: Shift values are mean ± one standard deviation. *N* = 10.

Abbreviation: CBCT, cone beam computed tomography

### 2.2 Image acquisition and reconstruction

For reference simulation CT (SimCT) images, 4DCT scans of the phantom were acquired for each breathing pattern using a surface‐guided motion tracking system (Vision RT, London, UK). The SimCT images for each breathing pattern were imported into the Ethos treatment planning system, and dummy plans were created to enable CBCT image acquisition.

The phantom was positioned on the treatment couch and aligned using the stationary external phantom geometry to eliminate any initial setup deviations. CBCT scans were performed using the 6‐second iCBCT Acuros mode, which utilizes the Acuros reconstruction algorithm that employs the Linear Boltzmann Transport Equation solving techniques to model and correct for scatter and associated artifacts, enhancing Hounsfield Unit (HU) accuracy and overall image quality [9]. Each respiratory signal was programmed to run for a duration of 3–4 min in a continuous loop to ensure consistency across multiple imaging acquisitions. The 4D SimCT (∼60 seconds) and CBCT (6 seconds or 60 seconds) scans were acquired starting at random points during the breathing cycle, simulating the variability inherent in clinical scenarios. For regular breathing patterns, three 6‐second CBCT scans were acquired. In the case of irregular breathing patterns, ten 6‐second CBCT scans were performed to account for the greater variability inherent in these conditions. Additionally, conventional 60‐second CBCT scans, also utilizing the Acuros reconstruction algorithm, were obtained to provide a comparative baseline for evaluating the performance of the 6‐second CBCT.

### 2.3 Image registration

Rigid registrations of the HyperSight CBCT images to the SimCT images were performed manually by a physician using the lung window setting. To minimize bias in decision‐making, the images were zoomed in to focus solely on the target, excluding external structures of the phantom. As the ETHOS couch does not support rotational corrections, only translational shifts were applied. These shifts refer to the displacement made by the physician during the image registration process. Given that the target motion was one‐dimensional along the superior‐inferior direction, only sagittal shifts were made.

For regular breathing patterns, the 4DCT average reconstruction SimCT images with the corresponding breathing patterns were used as the reference image set. However, for irregular breathing patterns, a SimCT scan acquired with a regular breathing pattern (15 breaths per minute [4‐s period] and 13 mm amplitude) was used as the reference. This approach reflects clinical practice, where patients with irregular breathing are guided to breathe regularly during 4DCT acquisition, and scans are repeated if the recorded breathing pattern during the scan is excessively irregular, potentially compromising the accuracy of the 4DCT scan.

Additionally, for the two most highly irregular breathing patterns, a second set of registrations was performed against 4DCT acquired with the corresponding irregular breathing patterns. This was done in recognition of the fact that patients with highly irregular breathing may have difficulty maintaining a regular breathing pattern during SimCT acquisition despite training and guidance, possibly due to the severity of their condition. This dual‐reference approach provided a comprehensive evaluation of the HyperSight system's ability to accurately capture target motion under both consistent and highly variable respiratory conditions.

A nonparametric unpaired *t*‐test was performed for irregular breathing patterns to assess the statistical significance of difference in shifts made with 6‐second and 60‐second CBCT scans. Additionally, the root‐mean‐square error (RMSE) was calculated to quantify the variability in shifts; $\mathrm{RMSE}=\sqrt{\frac{1}{n}\sum_{i=1}^{n}{\left(x_{i}-\mu \right)}^{2}}\;$, where $x_{i}$ represents each individual measured shift and 𝜇 is the mean shift for a given breathing pattern.

## 3 RESULTS

**FIGURE 2:**
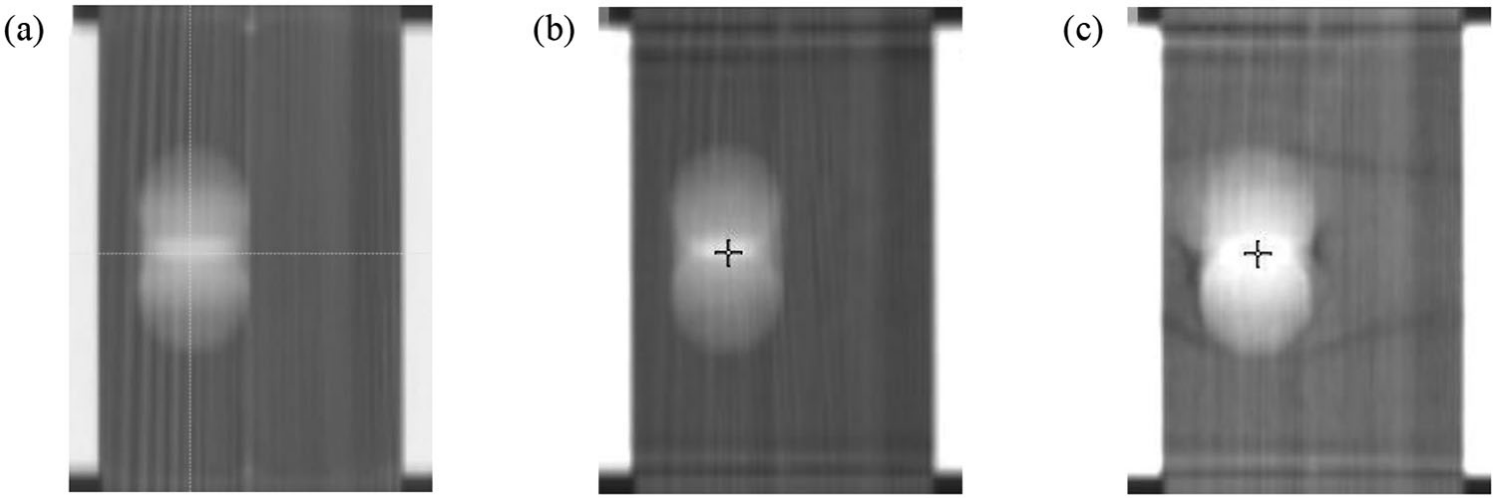
The spherical target imaged with (a) Sim CT, (b) 60‐second CBCT, and (c) 6‐second CBCT for a regular breathing pattern with a 4‐s period and 13 mm amplitude.

For the regular breathing patterns, the 6‐second CBCT scans demonstrated minimal shifts when compared to the SimCT images. (Figure 2) The average shift observed across all nine regular patterns was ‐0.2 ± 0.3 mm for the 6‐second CBCT, indicating high accuracy in target localization. Notably, the shifts were all less than 1.0 mm across all amplitudes and respiration rates. Equivalent results were observed with the 60‐second CBCT, which showed an average shift of −0.1 ± 0.3 mm (Table 1). The RMSE were ≤0.5 mm for all 6‐second CBCT scans, indicating high reproducibility despite different respiration rates and amplitudes. These findings suggest that the 6‐second CBCT of the HyperSight system reliably captures target motion under regular respiratory conditions, without compromising accuracy when compared to the traditional 60‐second CBCT.

The irregular breathing patterns presented more variability in the shifts observed during registration. Most shifts were within a clinically acceptable PTV margin of 5.0 mm. For 6‐second CBCT images, 7 out of 50 shifts (14%) exceeded 5.0 mm, all of which occurred in the highly irregular patterns. For moderately irregular patterns and the gasp/cough scenario, all shifts were within 5 mm, with overall averages of −1.3 ± 2.1 mm (range −0.7–3.6 mm) and −0.3 ± 1.1 mm (range −4.6–0.0 mm), respectively (see Table 2). Due to the inherently greater range of fluctuation, the two highly irregular patterns exhibited more variation in shifts, with an overall average of 3.9 ± 2.2 mm (range 0.0–8.1 mm), indicating potential challenges in accurately capturing target motion under these conditions. However, when the SimCT scans with corresponding irregular breathing patterns were used as the reference for image registration, the shifts were reduced, with an average of 1.7 ± 2.2 mm (range −2.3–5.1 mm). Only 1 out of 20 shifts (5%) exceeded the 5.0 mm PTV margin under these conditions.

**FIGURE 3:**
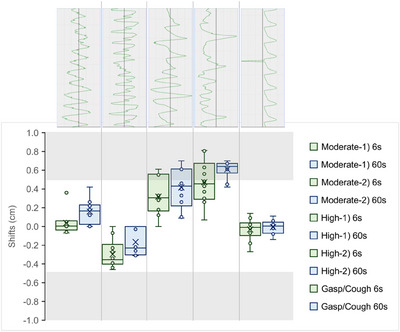
Shifts made from rigid image registrations for irregular breathing patterns. Shaded areas indicate shifts larger than the PTV margin of 5.0 mm.

**FIGURE 4:**
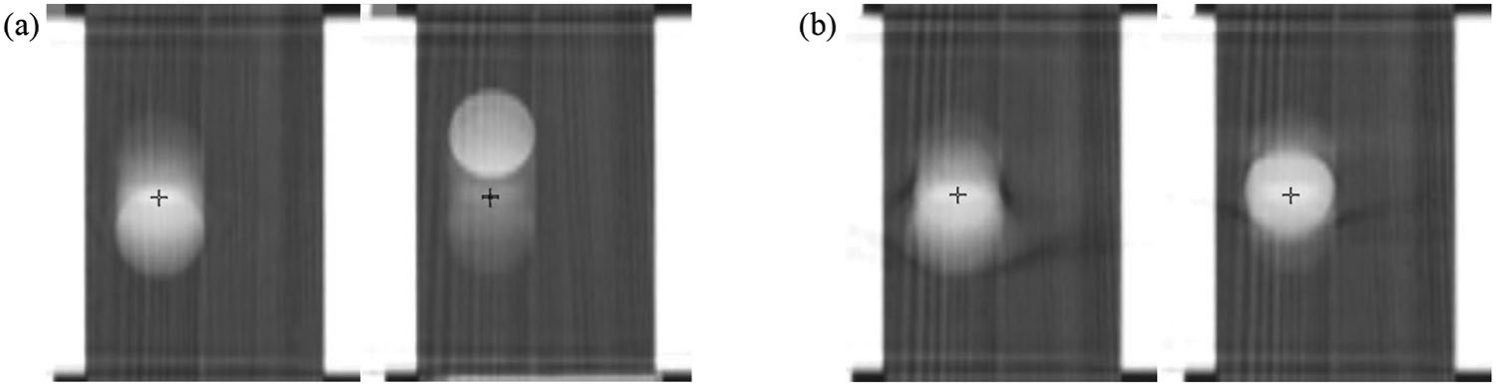
Variations of target location in CBCTs for a highly irregular breathing pattern. (a) 60‐second (b) 6‐second CBCTs.

The traditional 60‐second CBCT scans, used as a baseline for comparison, also demonstrated variability under irregular breathing conditions, with 12 out of 50 shifts (24%) exceeding 5.0 mm. Notably, all shifts greater than 5.0 mm occurred in the highly irregular patterns, while all shifts for the moderately irregular patterns and the gasp/cough scenario remained within the PTV margin, consistent with the results observed for the 6‐second CBCT under the same conditions (Figure 3). Figure 4 shows variations in captured target location in 6‐second and 60‐second CBCTs taken at two random time windows for the same highly irregular breathing pattern. Additionally, unpaired nonparametric *t*‐test returned *p*‐values ≥0.05 for the shifts made for 6‐second and 60‐second CBCT scans across all five irregular breathing patterns, indicating no statistically significant differences in shifts between the two scan modes (Table 2). RMSE values similarly reflected the increased variability associated with the degree of irregularity in breathing patterns. For the moderately irregular patterns and the gasp/cough scenario, RMSE remained well below the PTV margin of 5 mm, indicating clinically acceptable consistency despite breathing irregularities. However, in the highly irregular patterns, RMSE values were relatively higher than the regular breathing pattern dataset, especially when registered to regular breathing reference images. This consistency suggests that the challenges posed by highly irregular breathing patterns exist independently of the duration of the CBCT scan.

## 4 DISCUSSION

The traditional 60‐second CBCT acquisition provides the advantage of averaging out tumor motion, yielding a comprehensive view of the tumor's movement cycle. This comes at the cost of increased motion‐induced artifacts, which can degrade image quality and compromise the accuracy of patient setup verification. The fast CBCT on the HyperSight system offers a potential solution to this issue, but its reliability in capturing the accurate location of moving targets needed validation. This study demonstrates that the 6‐second CBCT can localize tumors with high accuracy under regular respiratory conditions, comparable to the traditional 60‐second CBCT. Despite initial concerns that the shorter imaging duration might limit the system's ability to capture the full range of target motion, our results indicate that the 6‐second CBCT performs robustly across a spectrum of respiratory conditions. For regular breathing patterns, it exhibited minimal shifts, with average deviations well within a typical PTV margin of 5.0 mm, even for a breathing period as long as 7.5‐s. These findings confirm the feasibility of using the HyperSight 6‐second CBCT for lung IGRT setup verification, where minimizing motion artifacts is crucial for precise radiation delivery.

It is important to acknowledge the minor distortions in target shape observed during the 6‐second CBCT scans, which are likely due to the limited number of projections inherent in shorter scan times (Figure 2). However, these distortions did not significantly impact the system's ability to accurately reconstruct the full range of target motion. Further study may be warranted for highly irregularly shaped targets, as this investigation is limited to a spherical phantom target that is presumably the simplest to reconstruct.

**FIGURE 5:**
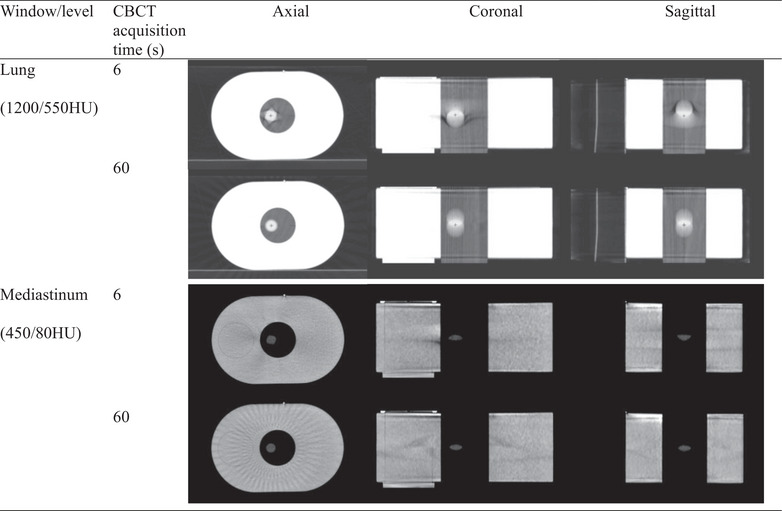
Motion artifact in 60‐second and 6‐second CBCTs for the respiratory motion phantom with a moderately irregular breathing pattern.

**FIGURE 6:**
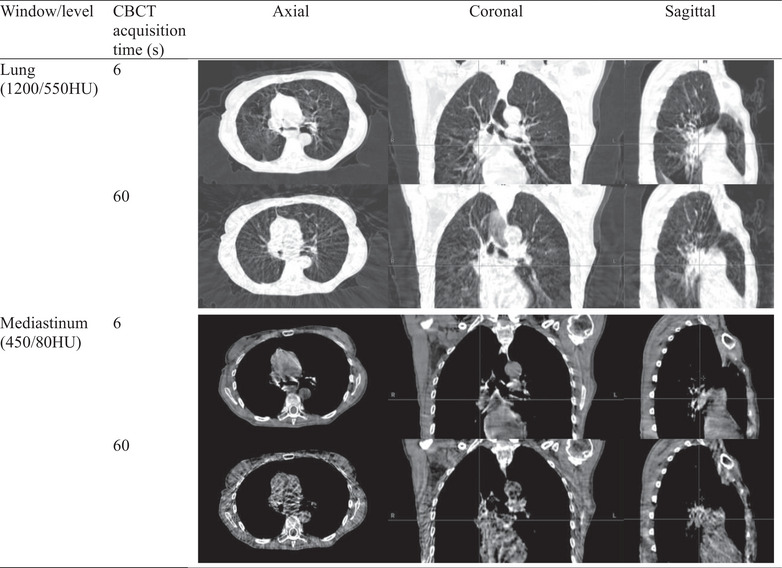
Motion artifact in 60‐second and 6‐second CBCTs for the same lung patient.

Despite minor distortions in target shape due to the limited number of projections, the 6‐second CBCT provided improved visualization for lung IGRT patient setup by reducing motion artifacts compared to the 60‐second CBCT. Figure 5 illustrates this effect in a phantom study, where the 6‐second CBCT exhibited reduced motion blur compared to the 60‐second scan. Despite both scans utilizing the same Acuros reconstruction algorithm, the 6‐second CBCT produced clearer images with significantly reduced motion artifacts compared to the 60‐second CBCT. This reduction in motion artifacts can be attributed to the shorter scan duration of the 6‐second CBCT, which limits the timeframe during which patient movement might occur, thereby minimizing the potential for image degradation from inconsistencies in acquired projection data. The difference in motion artifacts is more pronounced in patients, where surrounding anatomies are more complex and not stationary. Figure 6 presents a clinical example, comparing 60‐second and 6‐second CBCT scans for the same lung cancer patient acquired on the same day. The shorter imaging time of HyperSight 6‐second CBCT effectively “freezes” the patient's anatomy, capturing a more accurate representation of the tumor and surrounding tissues with minimal interference from motion. Since patient movement is largely restricted by immobilization devices, the predominant source of motion in lung IGRT is respiratory motion, which repeats cyclically throughout CBCT acquisition. Thus, while 60‐second CBCT captures a longer imaging period, it does not necessarily provide a more accurate representation of the tumor's motion range. Instead, the prolonged scan duration increases motion artifacts, making tumor localization more challenging. This improved visualization with the fast CBCT is particularly beneficial for IGRT patient setup, where patient motion, such as breathing or involuntary movements, can compromise the quality of longer scans.

The selection of breathing patterns in this study was based on clinical relevance, focusing on the range of respiratory cycles commonly observed in lung cancer patients. This study aimed to assess the feasibility of 6‐second CBCT for the majority of cases encountered in clinical practice rather than prioritizing uncommon scenarios. The selected breathing cycles (3.0–7.5 seconds) encompass the typical range seen in patients, whereas cycles exceeding 10 seconds are relatively rare. While evaluating the system's limitations is valuable, the primary objective was to determine its practical utility in routine IGRT workflows. Additionally, irregular breathing patterns, which present a more frequent clinical challenge than stable but prolonged breathing cycles, were included to reflect real‐world patient variability. These findings suggest that 6‐second CBCT is suitable for most patients, with additional caution warranted for those exhibiting highly irregular or exceptionally long breathing patterns.

The study also highlights the system's performance under more challenging conditions, such as highly irregular breathing patterns. While the 6‐second CBCT generally maintained accuracy, the increased variability in shifts observed during the two highly irregular breathing patterns suggests potential limitations (Figure 3). This issue was not unique to the 6‐second CBCT; the increased variability in shifts was also observed in the 60‐second CBCTs. This indicates that the uncertainty can be attributed to the significant unpredictability of the breathing patterns rather than the shortened acquisition time. Therefore, careful consideration should be given to patients with highly irregular breathing patterns, and efforts should be made to guide them towards more regular breathing patterns to ensure treatment accuracy, regardless of the CBCT scan duration.

While this study demonstrates the feasibility of using 6‐second CBCT for lung IGRT setup, several limitations should be acknowledged. First, although a range of clinically relevant respiratory patterns was tested, patient breathing can exhibit intrafractional variability and hysteresis effects, which were not explicitly modeled. Second, prolonged breathing cycles exceeding 7.5‐s were not included in this study due to their rarity in clinical practice; however, their occurrence is not negligible and posees potential risks of tumor misalignment. Zhao et al. ^[14^ ^]^. investigated respiratory periods up to 12‐s and found that excessively long breathing cycles could lead to target misalignment, likely due to the limited scan duration insufficiently capturing the full tumor motion range. Caution should be used for patients with prolonged respiratory cycles, and 60‐second CBCT should be considered, as these patients may not be ideal candidates for 6‐second CBCT. Lastly, while the 3 cm spherical target used in this study reflects typical lung tumor sizes of 2–4 cm ^[^ ^25^, ^26^, ^27^ ^]^, it does not fully represent the diverse tumor morphology that may affect registration accuracy and image visibility, particularly when reconstructed with limited projection data from the fast CBCT setting. Evaluating targets of various sizes and shapes could further generalize the results.

Future research could focus on further refining the HyperSight system's capabilities in handling irregular breathing patterns, potentially through advanced motion management techniques such as surface guidance. Exploring the use of the 6‐second CBCT in combination with other advanced imaging techniques may offer new avenues for improving the precision of IGRT in challenging clinical scenarios.

## 5 CONCLUSION

The fast acquisition CBCT of the HyperSight system demonstrated consistent and accurate performance in simulated lung IGRT setup verification, capturing tumor motion without compromising setup precision compared to the traditional 60‐second CBCT. Despite minor distortions in target shape due to the limited projections, the system successfully reconstructed the full range of target motion in various breathing scenarios. These findings validated the clinical utility of the 6‐second CBCT. While caution is warranted in cases involving highly irregular or exceptionally long breathing patterns, the overall performance of the 6‐second CBCT supports its adoption in clinical practice for normal breathing patients, with demonstrated localization utility comparable to the standard 60‐second CBCT acquisition for free‐breathing lung patients.

## AUTHOR CONTRIBUTIONS

Jihye Koo performed data acquisition, analysis, and drafted the manuscript. Jacqueline Andreozzi supervised the project, designed the study framework, data analysis, and manuscript preparation. Gage Redler contributed to study design, data analysis, and manuscript revision. Emily Keit contributed to data collection and manuscript revision. Vladimir Semenenko and Stephen A. Rosenberg contributed to the interpretation of the data and manuscript revision.

## CONFLICT OF INTEREST STATEMENT

The authors declare no conflicts of interest.
